# Treatment of *Bacillus cereus* endophthalmitis with endoscopy-assisted vitrectomy

**DOI:** 10.1097/MD.0000000000008701

**Published:** 2017-12-15

**Authors:** Qintuo Pan, Yanhua Liu, Ruixi Wang, Tianyu Chen, Zhengwei Yang, Yuxuan Deng, Zhenquan Zhao, Xuting Hu, Xiaomeng Chen, Wenlong Wei, Zongduan Zhang, Yuqin Wang, Jingwei Zheng, Zhisheng Ke

**Affiliations:** a The Eye Hospital of Wenzhou Medical University, Wenzhou, China; b Australian College Of Optometry Melbourne, Victoria, Australia.

**Keywords:** *Bacillus cereus*, endophthalmitis, endoscope, vitrectomy

## Abstract

To evaluate the use of endoscopy-assisted vitrectomy in patients with sight-threatening *Bacillus cereus* endophthalmitis.

A retrospective analysis was conducted in 15 eyes with *Bacillus cereus* endophthalmitis. Patients were divided into 2 groups: endoscopy-assisted vitrectomy (5 eyes) and conventional vitrectomy (10 eyes). The following clinical data were recorded and analyzed: sex, age, latent period, symptom duration, follow-up time, visual acuity pre- and postsurgery, recurrence of endophthalmitis, incidence of phithisis bulbi, and incidence of enucleation.

In the conventional vitrectomy group, postoperative visual acuity ranged from no light perception in 5 patients (50%), light perception in 3 patients (30%), 20/1000 in 1 patient (10%), and 20/50 in 1 patient (10%). In the endoscopy-assisted vitrectomy group, postoperative visual acuity ranged from no light perception in 2 patients (40%), light perception in 1 patient (20%), and hand movements in 2 patients (40%). There was no statistically significant difference between the 2 groups in terms of the final postoperative visual acuity (*F* = 0.006, *P* = .937). There is no difference between the 2 groups in terms of the incidence of enucleation. The median symptom duration was 4 hours (range: 2–6 hours) in the conventional group and 9 hours (range: 7–11 hours) in the endoscopy-assisted vitrectomy group. The difference in the symptom duration between the 2 groups was statistically significant (*P* = .002).

There is no statistical significant difference between the 2 groups in terms of visual acuity and incidence of enucleation. Therefore, endoscopy-assisted vitrectomy can be considered as an alternative treatment for treatment of *B cereus* endophthalmitis particularly for cases when symptom duration was more than 6 hours.

## 1 Introduction

Endophthalmitis is defined as inflammation within the anterior or posterior segment, often involving the vitreous and/or the aqueous humour. Endophthalmitis is commonly caused by bacterial or fungal infections, which often leads to partial or complete loss of vision. Bacterial endophthalmitis usually results from infection of the posterior segment following intraocular surgery (postoperative), penetrating eye injury (post-traumatic), or from septic spread of infection (endogenous).^[1,2]^ The incidence of endophthalmitis following a penetrating eye injury varies from 2 to 17%.^[3,4]^ Patients with endophthalmitis often present with severe ocular pain, periorbital swelling, corneal ring abscess, proptosis, and fever.^[5–7]^ Although *Bacillus cereus* (*B cereus*) endophthalmitis is rare, it is the second most frequent causes of post-traumatic bacterial endophthalmitis. *B cereus* endophthalmitis is unique in its rapid time course and delays in treatment will result in permanent vision loss. In severe cases of endophthalmitis with longer symptom duration, it can lead to the devastating consequence of phthisis bulbi. Phthisis bulbi represents an ocular end-stage disease characterized by atrophy, shrinkage, and disorganization of the eye and intraocular contents.^[8]^

The most important aspect of treatment is intravitreal injection of antibiotics, along with vitrectomy in severe cases. However, conventional vitrectomy can be difficult to perform in some cases of *B cereus* endophthalmitis, which are complicated by poor visibility through the anterior segment with the potential for damaging intraocular structures during the surgery. An ophthalmic endoscope has the potential to overcome the limitations of poor visualization.^[9–11]^ It can enhance the visualization of the posterior segment, permitting direct assessment of retinal integrity and allowing the surgeon to perform vitrectomy safely and completely. It is also possible to handle postoperative complications such as dropped nuclei and foreign bodies more easily.^[12,13]^

The purpose of this study is to investigate the use of an endoscopic approach in the treatment of *B cereus* endophthalmitis. To our knowledge, this is the first study to investigate the ocular endoscope approach in the treatment of *B cereus* endophthalmitis.

## 2 Materials and methods

Fifteen eyes of 15 patients with *B cereus* endophthalmitis were admitted to the Eye Hospital of Wenzhou Medical University, China from December 2010 to January 2016. The minimum follow-up time was 6 months. This study followed the Declaration of Helsinki and ethical approval was obtained from the Ethics Committees in Eye Hospital of Wenzhou Medical University. Inclusion criteria were infective endophthalmitis caused by penetrating wounds, and evidence of *B cereus* growth on the culture of vitreous or aqueous humor. Exclusion criteria were the presence of a pre-existing retinal detachment extending into the central visual axis, any history of other ocular injury, severe systemic diseases, and other infective diseases.

**Figure 1:**
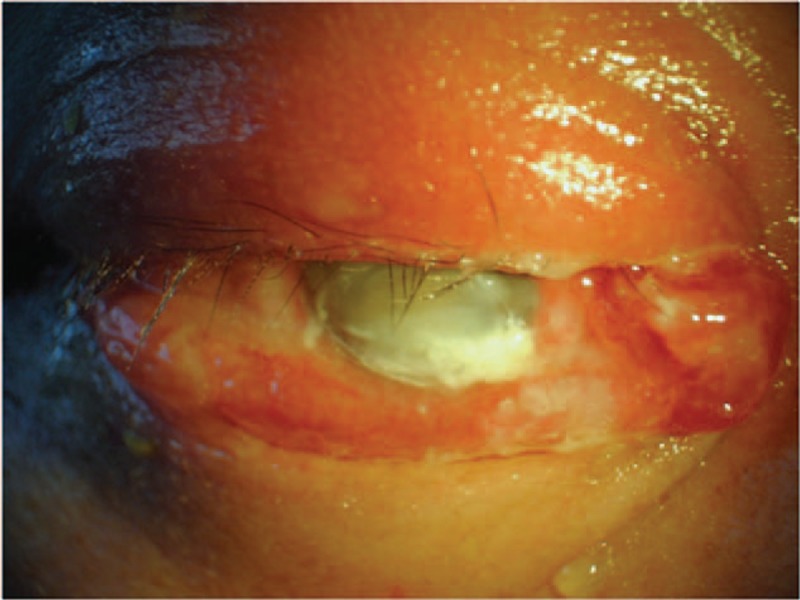
Colored photograph of the external eye in a 48-year-old male patient who has developed *B cereus* endophthalmitis after penetrating trauma. The figure demonstrates the following clinical signs: corneal opacification, chemosis, periorbital swelling, corneal ring abscess, and proptosis. This patient had preoperative visual acuity of light perception and his infection was controlled with endoscopy-assisted vitrectomy combined with silicon oil tamponade. His visual acuity was hand movements at the final follow-up of 59 months postoperatively.

Patients received 1 out of the 2 possible procedures: conventional vitrectomy (CV) or endoscopy-assisted vitrectomy (EAV). The decision to perform EAV is based on the presence and degree of corneal opacification (Fig. 1), in which visualization through the anterior segment was compromised for CV to be performed successfully, completely, and safely. Ten out of 15 patients underwent conventional vitrectomy and 5 patients underwent endoscopy-assisted vitrectomy.

All patients had not received any previous intraocular surgery for the treatment of their ocular trauma except 1 patient, who had received suture debridement and vitreous injection with vancomycin (0.1 mL of 1 mg/0.1 mL) and ceftazidime (0.1 mL of 1 mg/0.1 mL) prior vitrectomy.

The following patient characteristics were recorded: sex, age, mode and time of trauma, species, latent period (interval between the initial trauma and onset of symptoms of endophthalmitis), symptom duration (interval between onset of symptoms of endophthalmitis and when vitrectomy was conducted), follow-up time, visual acuity before surgery and at the last follow-up, surgical procedures and ocular outcomes. All patients had slit lamp examinations, and B-scan ultrasonography pre- and postoperatively.

The decision to have general versus topical anesthesia was based solely on the patient's preference and their pain tolerance.

## 3 Conventional vitrectomy (CV) procedures

After the administration of general or topical anesthesia, all patients underwent standard three-port pars plana vitrectomy. Three incisions were created at the pars plana and the infusion cannula was inserted into the inferotemporal cannula. Before opening the infusion catheter, a small core sample of the vitreous (0.2 mL) and aqueous humor (0.1 mL) was extracted for bacterial/fungal culture and bacteria/fungus sensitivity test and smear. The lens and capsule were removed during the surgery. All of the visible vitreous gel and inflammatory debris were removed as complete as possible using the Millennium 23-gauge high-speed vitrector, while continually supplying balanced salt solution (BSS) as irrigating solution to maintain the intraocular pressure. Silicone oil was used as the tamponade agent after laser treatment or cryotherapy in 8 cases with co-existing retinal detachment or retinal tear which did not extend to the central visual axis.

## 4 Endoscopy-assisted vitrectomy (EAV) procedures

After the administration of general or topical anesthesia, all patients underwent standard three-port pars plana vitrectomy. The endoscope probe (Endognost CS200, POLYDIAGNOST GMBH, Germany) was inserted into the eye through the pars plana by an operating microscope. Under endoscopic visualization with manual suction and the infusion catheter closed, the lens and capsule were removed and a small sample of the vitreous (0.2 mL) and aqueous humor (0.1 mL) was extracted for bacterial/fungal culture and sensitivity test and smear. After the infusion catheter was turned on, a vitrectomy cutter was inserted through another sclerotomy site as in the conventional pars plana vitrectomy. The vitreoretinal procedures were performed as usual with the sole difference being that the retina is viewed on the monitor near the surgeon instead of through the microscope. Silicone oil was used as the tamponade agent after laser treatment or cryotherapy in 4 cases with co-existing retinal detachment or retinal tear.

At the end of CV and EAV procedures, the vitreous was infused with vancomycin (0.1 mL of 1 mg/0.1 mL) and ceftazidime (0.1 mL of 1 mg/0.1 mL).

We have defined the treatment of endophthalmitis as “successfully controlled” when there was no recurrence of endophthalmitis, and ocular structures were preserved without the need of enucleation.

Statistical analysis was performed using SPSS 19.0 software (SPSS Inc.). Data that departed from a Normal distribution were described and analyzed using nonparametric statistics. The difference between the 2 groups was assessed using the nonparametric Mann–Whitney *U* test. Covariance analysis and Fisher's exact test were used to evaluate the difference in visual and ocular prognosis between the 2 groups. In all analyses, *P* < .05 was considered to be statistically significant.

## 5 Results

Fifteen patients (14 males, 1 female) were included in the study. Table 1 displays the clinical data recorded for each patient. Cultures were positive of *B cereus* in 15 patients. Table 2 demonstrates the clinical data when compared between the 2 treatment groups. Ten patients underwent conventional pars plana vitrectomy (CV group), and 5 patients underwent endoscopy-assisted vitrectomy (EAV group). There were no intraoperative complications.

**Table 1:**
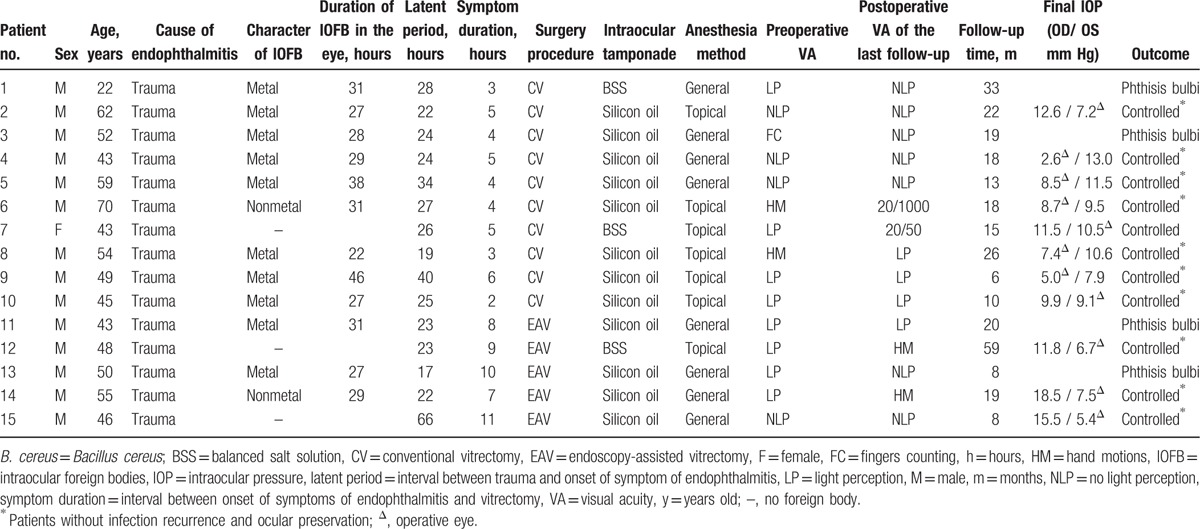
Data for patients with *B cereus* endophthalmitis treated with conventional or endoscopy-assisted vitrectomy.

**Table 2:**
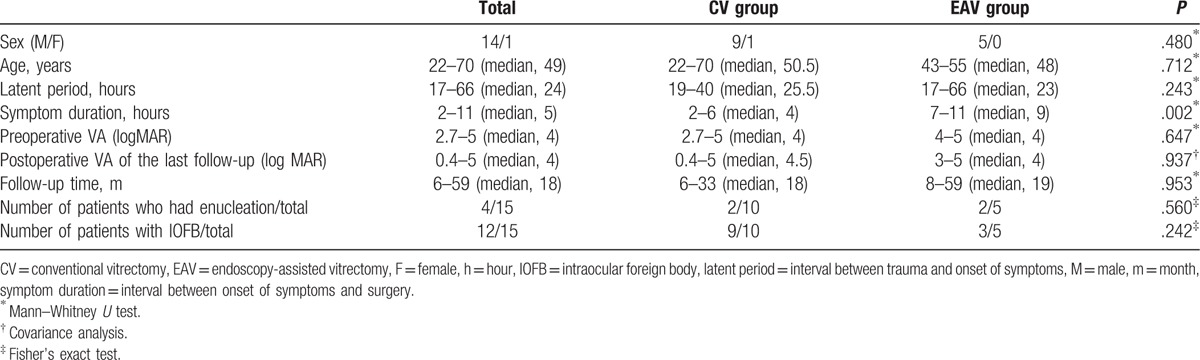
Characteristics of groups treated with conventional vitrectomy or endoscopy-assisted vitrectomy.

In the CV group, four patients (40%) underwent general anesthesia and 6 patients (60%) underwent topical anesthesia. Preoperative visual acuity ranged from no light perception to count fingers. Postoperative visual acuities were no light perception in 5 patients (50%), light perception in 3 patients (30%), 20/1000 in 1 patient (10%), and 20/50 in 1 patient (10%). The symptom duration ranged from 2 hours to 6 hours (median, 4 hours). The infection is described as successfully controlled in 8 patients (80%) with a median intraocular pressure (IOP) of 7.95 mm Hg (range: 2.6–10.5 mm Hg) at the final follow-up. The remaining 2 patients (20%) had phthisis bulbi which required enucleation for better cosmetic appearance.

In the CV group, 8 patients had silicon oil tamponade at the end of the surgery. One of the 8 patients developed proliferative vitreoretinopathy (PVR) postoperatively and had a secondary vitrectomy surgery with silicon oil refilled. The other 7 patients did not undergo silicon oil removal, three patients refused silicon oil removal due to poor visual outcome, 2 patients with partial retinal detachment refused further treatment, and 2 patients with low IOP kept silicon oil in the vitreous to maintain the shape of the eye. Two patients did not have silicone oil tamponade at the end of the surgery, one of these 2 patients required enucleation and the other one retained the best vision of 20/50.

In the EAV group, 4 patients (80%) underwent general anesthesia and 1 patient (20%) underwent topical anesthesia. Preoperative visual acuity ranged from light perception to count fingers. Postoperative visual acuity ranged from no light perception in 2 patients (40%), light perception in 1 patient (20%), and hand movements in 2 patients (40%). The symptom duration varied from 7 hours to 11 hours (median, 9 hours). The infection is described as successfully controlled in 3 patients (60%) with silicon oil tamponade with a median IOP of 6.7 mm Hg (range: 5.4–7.5 mm Hg) at the final follow-up. The other 2 patients (40%) had outcome of phthisis bulbi which required enucleation for better cosmetic appearance.

In the EAV group, 4 patients had silicon oil tamponade at the end of the surgery. Two out of the 4 patients refused silicon oil removal due to poor visual outcome and 1 out of the 4 patients with low IOP kept silicon oil in the vitreous to maintain the shape of the eye. The other patient had complication of proliferative vitreoretinopathy and had a secondary vitrectomy surgery with silicon oil refilled.

There was a statistically significant difference between the 2 groups in symptom duration (*P* = .002). There was no statistically significant difference between the 2 groups in terms of patient's sex (*P* = .480), patient age (*P* = .712), patient period (*P* = .243), preoperative (*P* = .647), and postoperative visual acuity (*P* = .937), follow-up time (*P* = .953), the number of patients with intraocular foreign bodies (*P* = .242), and the incidence of enucleation (*P* = .560).

## 6 Discussion

*B cereus* has been implicated as a major pathogen responsible for post-traumatic endophthalmitis, and has the potential to cause a fulminant infection with devastating visual consequences.^[14]^ Previous studies have shown that *B cereus* endophthalmitis develops faster than endophthalmitis caused by other gram-positive pathogens such as *Streptococcus pneumoniae*,^[15,16]^*Staphylococcus aureus*,^[17,18]^ or *Enterococcus faecalis*.^[19,20]^ This is attributed to the virulence of the organism. In cases of post-traumatic endophthalmitis caused by *B cereus*, patients may begin to experience sudden onset of severe ocular pain within 18 to 24 hours after ocular trauma and may demonstrate clinical signs including chemosis, periorbital edema, proptosis, and peripheral corneal edema.^[7]^ This is consistent with our study where the onset of symptoms for *B cereus* endophthalmitis is between 17 and 66 hours (median, 24 hours) post-trauma and 66.67% (10/15) patients had a latent period within 24 hours.

Many previous reports have shown that the outcomes are poor for cases with post-traumatic *B cereus* endophthalmitis, often leading to complete vision loss with limited potential for restoration of useful vision. Some cases require enucleation of the eye as a result of phthisis bulbi.^[7,21,22]^ In our study, at the final postoperative follow-up, 46.7% of patients (7/15) had no light perception, only 6.7% (1/15) had a visual acuity of 20/50 and the rest of patients had visual acuity ranging from light perception to 20/1000. Despite appropriate surgical intervention, 2 patients (20%) in CV group and 2 patients (40%) in EAV group had phthisis bulbi which required enucleation for better cosmetic appearance. This highlights the importance of extensive research for improved treatment strategies for ocular condition. The patient with the best postoperative visual acuity of 20/50 may be explained by relatively early surgical intervention and timely administration of intravitreal antibiotics prior to vitrectomy.

In an experimental model of *B cereus* endophthalmitis in rabbit eyes, intravitreal injection of vancomycin achieved complete sterilization in infected eyes when it was administered as late as 6 hours postinfection.^[23]^ However, time courses of these infections may not have been similar to that seen in human infections.^[24,25]^ In another experimental model of *B cereus* endophthalmitis,^[26]^ the efficacy of vitrectomy combined with vancomycin was more effective than that of vancomycin alone for limiting inflammation and salvaging vision. In this model, 4 hours postinfection was the critical time within which intravitreal vancomycin must be initiated to salvage useful vision.

The Endophthalmitis Vitrectomy Study recommended vitrectomy as the best therapeutic option for cases with visual acuity of hand motion or worse.^[27]^ Vitrectomy can remove potentially harmful contents and pathogens from the inside of the eye to minimize inflammation and salvage vision for many types of ocular infections.^[28–30]^ However, conventional vitrectomy is difficult to perform for cases with evidence of anterior segment opacification. This is particularly relevant for *B cereus* endophthalmitis due to the rapid progression of the disease and higher risk of corneal opacification.

In addition, vitrectomy should be administered in a timely manner when there is limited improvement in vision with intravitreal antibiotics alone. However, delay in vitrectomy may become inevitable in certain cases. Due to the severity of ocular manifestations of *B cereus* endophthalmitis, some patients need to have general anesthesia as topical anesthesia cannot be tolerated. However, general anesthesia requires fasting which prolongs surgical preparation time and may lead to a delay in the administration of vitrectomy in a timely manner. This is evident by our study, where patients who could not tolerate topical anesthesia had a longer duration of symptoms prior to the intervention. In the EAV group, most cases (80%) had general anesthesia with a symptom duration that ranges between 7 and 11 hours (median, 9 hours), which is longer than the CV group. The difference between the 2 groups is statistically significant (*P* = .002). Therefore, the use of general anesthesia will inevitably delay vitrectomy and indirectly worsens the ocular condition, predisposing patients to sight-threatening consequences including corneal opacification or disorganized structures of the anterior segment. It also adds difficulty to vitrectomy using the conventional viewing system with an operating microscope due to impeded visualization of the posterior segment.

In cases with dense corneal opacification that impedes the visualization of the posterior segment with conventional vitreoretinal intervention, there are several treatment approaches, including observation of the posterior segment until the cornea clears with medical management, performing a conventional vitrectomy combined with keratoprosthesis,^[31]^ or endoscopic-assisted vitrectomy.^[32]^ The selection of the approach depends on the urgency of the intervention and availability of corneal grafts. In patients with post-traumatic endophthalmitis, there is a higher risk of graft failure, and regrafting also carries a higher risk of graft rejection.^[11]^ In addition, placing and removing the keratoprosthesis increases the duration of surgery.^[31,33]^ Therefore, endoscopy-guided vitrectomy can be considered to improve visual and anatomical outcome in patients with *B cereus* endophthalmitis.

Despite the significantly longer symptom duration of EAV group when compared to the CV group (*P* = .002), visual outcome measures were not significantly different between the 2 groups (*F* = 0.006, *P* = .937). This is attributed to some unique advantages of EAV when compared to conventional microscopic vitrectomy units. The advantages of the endoscopic system in vitreoretinal surgery are based on 2 principles: (1) bypassing opacities of the anterior segment; and (2) enhance the visualization of anterior structures, such as the ciliary bodies, posterior iris surface, pars plana, and peripheral retina.^[34]^ Endoscopy-assisted vitrectomy allows more controlled core vitrectomy and provides direct visualization of the ocular structures. This encourages the removal of the offending toxic contents in the central vitreous and inflammatory membranes under the iris and over the ciliary body,^[11]^ which leads to improved visual outcome. It can also distinguish normal retina from necrotic retina. Fibrous exudation on the posterior retina can simulate necrosis due to its firm attachment and white appearance.^[10]^

It is important to note that there is no statistically significant difference between the EAV and CV group in terms of the incidence of enucleation (*P* = .560). Even though the incidence of patients in the EAV group who had enucleation is double than the CV group, there is still no statistically significant difference between the 2 groups. There is a higher incidence of patients that required enucleation in the EAV group, this may be attributed to longer symptom duration of the condition, poorer preoperative visual acuity and hence guided postoperative prognosis.

In our study, most cases required silicone oil tamponade for retinal detachment or retinal tear which resulted from *B cereus* infection. Silicone oil tamponade is beneficial for maintaining the shape of intraocular structures and is known to possess antimicrobial activities,^[35]^ including eliminating *B cereus*.^[36]^

It is also important to note that endoscopy-assisted vitrectomy is a new surgical procedure which creates a learning curve that needs to be overcome. Due to the difference in the viewing systems between the conventional vitrectomy and endoscopic vitrectomy, endoscopic vitrectomy can be challenging, because surgeons will need to view a remote monitor while performing the operation. In addition, there is loss of stereopsis, therefore, awareness of the location of the instrument within the globe therefore relies on monocular clues such as the size of landmarks, intensity of illumination and changes in focus.^[11,37]^

There are several limitations in our study. For example, the sample size of the study is small with a relatively short follow-up time. The small sample size of the study is due to several factors. *B cereus* endophthalmitis is a relatively rare ocular condition in nature. The study has restricted in the inclusion criteria to only include *B cereus* endophthalmitis caused by penetrating wounds. In addition, we have carried out a retrospective analysis but not a prospective randomized controlled trial, which is the gold standard trial for evaluating the effectiveness of therapy. Therefore, we cannot conclude from this study that endoscopy-assisted vitrectomy is superior in efficacy when compared to conventional vitrectomy. In the future, prospective studies should be conducted to evaluate the effectiveness of endoscopy-assisted vitrectomy in a larger population.

In conclusion, EAV surgery can be considered as an alternative therapy for the treatment of sight-threatening *B cereus* endophthalmitis. It can enhance the visualization of the posterior segment of the eye in some cases of *B cereus* endophthalmitis, especially when symptom duration is more than 6 hours.
